# Head and Neck Cancer among American Indian and Alaska Native Populations in California, 2009–2018

**DOI:** 10.3390/cancers13205195

**Published:** 2021-10-16

**Authors:** Brooke R. Warren, Jennifer R. Grandis, Daniel E. Johnson, Alessandro Villa

**Affiliations:** 1 School of Medicine, University of California, San Francisco, CA 94143, USA; Brooke.Warren@ucsf.edu; 2 Department of Otolaryngology–Head and Neck Surgery, University of California, San Francisco, CA 94143, USA; Jennifer.Grandis@ucsf.edu (J.R.G.); Daniel.Johnson@ucsf.edu (D.E.J.); 3 Department of Orofacial Sciences, University of California, San Francisco, CA 94143, USA

**Keywords:** head and neck cancer, American Indian, human papilloma virus

## Abstract

**Simple Summary:**

In the United States, it is estimated there will be 54,000 new cases of oral cavity and pharyngeal cancer in 2021. Tobacco exposure and drinking alcohol are the main causes of head and neck cancer (HNC). Human Papilloma Virus (HPV) is now increasing in prevalence and is the most common cause of oropharyngeal cancer in the United States. This study assessed the incidence of HNC and HPV status in American Indian/Alaska Native (AI/AN) populations in California and determined if incidence was higher among AI/ANs compared to other ethnicities. We found that AI/AN and White patients had the highest burden of late-stage HNC and HPV+ lip, oral cavity, and pharynx cancer compared to other ethnicities. In addition, AI/ANs had a decreased survival rate compared to White patients. These findings reveal ethnic or racial differences in incidence, presentation, and survival, and should inform future preventative care measures for the AI/AN population.

**Abstract:**

The purpose of this study was to determine the incidence of HPV-positive (HPV+) and HPV-negative (HPV-) head and neck cancer (HNC) in the American Indian/Alaska Native (AI/AN) population in California to assess whether incidence is higher among AI/ANs compared to other ethnicities. We analyzed data from the California Cancer Registry, which contains data reported to the Cancer Surveillance Section of the Department of Public Health. A total of 51,289 HNC patients were identified for the years 2009–2018. Outcomes of interest included sex, stage at presentation, 5-year survival rate, tobacco use, and HPV status. AI/AN and White patients had the highest burden of late stage HNC (AI/AN 6.3:100,000; 95% CI 5.3–7.4, White 5.8:100,000; 95% CI 5.7–5.9) compared to all ethnicities or races (Black: 5.2; 95% CI 4.9–5.5; Asian/Pacific Islander: 3.2; 95% CI 3–3.3; and Hispanic: 3.1; 95% CI 3–3.2 per 100,000). Additionally, AI/AN and White patients had the highest burden of HPV+ lip, oral cavity, and pharynx HNC (AI/AN 0.9:100,000; 95% CI 0.6–1.4, White 1.1:100,000; 95% CI 1–1.1) compared to all ethnicities or races (Black: 0.8:100,000; 95% CI 0.7–0.9; Asian/Pacific Islander: 0.4; 95% CI 0.4–0.5; and Hispanic: 0.6; 95% CI 0.5–0.6). AI/ANs had a decreased 5-year survival rate compared to White patients (AI/AN 59.9%; 95% CI 51.9–67.0% and White 67.7%; 95% CI 67.00–68.50%) and a higher incidence of HNC in former and current tobacco users. These findings underscore the disparities that exist in HNC for California AI/AN populations. Future studies should aim to elucidate why the unequal burden of HNC outcomes exists, how to address increased tobacco usage, and HPV vaccination patterns to create culturally and community-based interventions.

## 1. Introduction

Cancer is the second leading cause of death for American Indian/Alaska Natives (AI/ANs) in the United States [1]. Cancer incidence rates vary by geographic region for AI/AN populations. The incidence rates of lung and colorectal cancers were reported to be three times higher in AI/AN males and females compared with White patients in the Great Plains, Pacific Coast, and Alaskan regions of the US. In all regions, cancers of the liver, kidney, and stomach were significantly higher in AI/ANs than White patients [2,3].

The incidence of head and neck cancer (HNC) among AI/AN populations is less well studied. It is estimated that in 2021, 54,000 new cases of oral cavity and pharyngeal cancer will occur in the United States, with 10,000 cancer-related deaths and a 5-year survival rate of 66% [4]. HNC has historically been linked to tobacco exposure, most commonly cigarette smoking, in conjunction with drinking alcohol. Persistent infection with high-risk strains of Human Papilloma Virus (HPV) is the most common cause of oropharyngeal cancers in the United States and there has been a continuous increase in the prevalence and incidence of HPV-positive (HPV+) HNC for the past 2–3 decades [5], with males being the most affected. Thus, HNCs not associated with HPV are now known as HPV-negative HNC.

While many studies have assessed the disparities in HNC rates in African American and Latinx populations compared to White populations [6,7], little is known regarding comparative HNC epidemiology in AI/ANs. In the Center for Disease Control’s (CDC) most recent national data from 2018, 265 new cases of oral cavity and pharynx cancer were reported among the AI/AN population as well as 50 AI/AN deaths from this cancer [8]. Two studies reported that the incidence rate of late-stage HNC increased 75.3% from 2004 to 2015 in AI/AN and Asian/Pacific Islanders [9,10]. The Institute of Population Health at the University of California Davis released data from the period of 2000–2016 showing that AI/ANs (aged 20–44 years) in California similarly had increased incidence of late-stage oral cancer diagnosis compared to White patients. This report also found that AI/ANs had a lower 5-year survival rate for patients with oropharynx cancers [11]. According to the Indian Health Service cancer registry, oropharyngeal cancers were the most common HPV+ cancer among AI/AN males in all regions of the United States (from 2013 to 2017) [1,11,12].

Few studies have examined HNC in the AI/AN population in California [13,14], the most populated state in the United States with 39.5 million people and 631,000 people identifying as American Indian [15]. Investigation of the HNC burden among AI/ANs will identify opportunities to improve prevention and treatment for this population. To this end, we determined the incidence of HNC and HPV status of patients in California according to sex and race or ethnic group to assess whether the incidence of HPV+ and HPV− HNC is higher among AI/AN compared to other ethnicities in this large, diverse state.

## 2. Materials and Methods

### 2.1. Study Population

We analyzed data from the California Cancer Registry (CCR) [16], a surveillance database containing cancer data from California medical centers reported to the Cancer Surveillance Section of the Department of Public Health. Patients were identified who were 18 years or older and had a diagnosis of HNC from 2009 to 2018. Cases diagnosed at VA hospitals were excluded due to VA restrictions with their submissions to central cancer registries. This study was approved by the University of California, San Francisco Institutional Review Board (IRB number: 21-33716).

### 2.2. Data Collection

We identified all cases of HNC (oral cavity, pharynx, larynx, lip, oropharynx, nasopharynx, hypopharynx, paranasal and accessory sinuses, and salivary glands) using the International Classification of Diseases for Oncology, 3rd edition [12]. The primary covariate of interest was AI/AN race as designated by the CCR database. Other races were classified as non-Hispanic White, non-Hispanic Black, Hispanic, and Asian or Pacific Islander. For conciseness, the term “non-Hispanic” was omitted when discussing groups in this study. The outcomes of interest were sex, age at cancer diagnosis, stage at presentation, cancer-specific mortality, 5-year survival rate, tobacco use, and HPV status. HPV status was collected as defined by the CS Collaborative Stage Data Collecting System, version 02.05 schemas [17]. HPV status was determined on pathologic specimens by immunohistochemistry for p16 expression and in situ hybridization, polymerase chain reaction (PCR), real time-PCR for viral DNA or RNA detection [18]. Grouped HPV status of the lip, oral cavity, and pharynx was available for the years 2009–2018. HPV status for individual sites within the pharynx (oropharynx, nasopharynx, hypopharynx), larynx, paranasal and accessory sinuses, and salivary glands HNC sites was limited to 2016–2018. The year 2016 represents the first-year data on HPV status for oropharynx, nasopharynx, hypopharynx, larynx, paranasal and accessory sinuses, and salivary glands were listed for HNC in the CCR.

### 2.3. Statistical Analysis

Age-Adjusted Rates in this report were calculated using the Surveillance Research Program, National Cancer Institute SEER*Stat software version 8.3.9. The yearly incidence and mortality per 100,000 persons for each age group were age-adjusted to the 2000 United States standard population. Rates based on less than five cases (or deaths) in any given year were not calculated [19]. We then calculated the 95% confidence intervals for each category of race or ethnicity, sex, age at cancer diagnosis, tobacco use, stage at diagnosis, cancer-specific mortality, 5-year survival rate, and HPV status.

## 3. Results

### 3.1. Overall Incidence, Age at Diagnosis, and Tobacco Use

Table 1 shows trends in the age-specific incidence of HNC (per 100,000 persons), including all races and ethnic groups, according to the calendar period from 2009 through 2018. A total of 51,289 patients (320 AI/AN and 50,969 non-AI/AN) with HNC were identified. White males had the highest HNC incidence rate (23.6:100,000; 95% CI 23.3–23.9) among all men, and White females had the highest rate (8.2:100,000; 95% CI 8–8.3) among all women. AI/ANs had the second highest incidence rate of HNC among men and women (males 22:100,000; 95% CI 19.1–25.1 and females 7.4:100,000; 95% CI 5.8–9.2, respectively), followed by Black patients (males 18.7:100,000; 95% CI 17.8–19.5, females 6.7:100,000; 95% CI 6.2–7.1), Asian/Pacific Islanders (males 13.5:100,000; 95% CI 13.1–14, females 5.6:100,000; 95% CI 5.3–5.8), and Hispanic patients (males 12.8:100,000; 95% CI 12.5–13.2, females 4.6:100,000; 95% CI 4.4–4.8).

For AI/ANs aged 40–49 and 80+, the incidence rate of HNC in these age groups (12.9:100,000; 95% CI 9.1–17.1 and 67.2:100,000; 95% CI 46.5–93.9) was highest compared to all ethnicities. Amongst current and former tobacco users, the incidence rate of HNC in AI/ANs was 5.0 per 100,000 compared to never used tobacco (2.2:100,000). In contrast, in White current and former tobacco users, the incidence rate of HNC was 4.4 per 100,000, and 2.4 per 100,000 for those who had never used tobacco.

### 3.2. Stage at Diagnosis, Mortality, and 5-Year Survival

In Table 2, when cancer stage was considered, AI/AN males and females had the highest incidence rate of stage IV HNC (6.3:100,000; 95% CI 5.3–7.4) compared to other ethnicities (White: 5.8; 95% CI 5.7–5.9; Black: 5.2; 95% CI 4.9–5.5; Asian/Pacific Islander: 3.2; 95% CI 3–3.3; and Hispanic: 3.1; 95% CI 3–3.2 per 100,000). Black HNC patients were found to have the highest all-cause mortality rate (4:100,000; 95% CI 3.8–4.3) compared to all ethnicities. Additionally, Black male and female HNC patients have the lowest 5-year survival rate (55.95%; 95% CI 53.30–58.40%) followed by AI/AN patients (59.9%; 95% CI 51.9–67.0%), with AI/AN females having the lowest 5-year survival rate (49.0%; 95% CI 31.0–64.7%).

### 3.3. HPV Status

In Table 3, the 10-year period from 2009 to 2018 includes a total of 25,914 patients with lip, oral cavity, and pharynx HNC who had HPV status reported (142 AI/AN and 25,772 non-AI/AN). The incidence of HPV+ lip, oral cavity, and pharynx cancer was highest in White patients (1.1:100,000; 95% CI 1–1.1) followed by AI/AN patients (0.9:100,000; 95% CI 0.6–1.4) and Black patients (0.8:100,000; 95% CI 0.7–0.9).

Between 2016 and 2018, a total of 7619 patients with HNC (lip, oral cavity, pharynx, hypopharynx, nasopharynx, oropharynx, soft palate, pharyngeal tonsil, tongue base, and other pharyngeal) had HPV status reported (45 AI/AN and 7574 non-AI/AN). The overall incidence for HPV+ HNC (Table 4) was highest in White patients (2.8:100,000; 95% CI 2.7–3), followed by AI/AN patients (2.7:100,000; 95% CI1.6–4.2). The incidence for HPV+ HNC was lowest for Asian/Pacific Islanders (0.6:100,000; 95% CI 0.5–0.7). HPV+ cancer of the tongue base was highest in White patients (1.1:100,000; 95% CI 1–1.2) and second highest in AI/AN (0.9:100,000; 95% CI 0.3–1.9). AI/AN patients had the highest HPV+ incidence rate for oropharyngeal cancers at 1.8 per 100,000 (95% CI 1–3.1).

### 3.4. HPV-Related Cancers

For the 10-year period from 2009 to 2018 (Table 5), AI/ANs had the highest age-adjusted incidence of cervical cancer (13:100,000; 95% CI 10.8–15.4) compared to Hispanic (9.1:100,000; 95% CI 8.9–9.4), Black (7.7:100,000; 95% CI 7.2–8.2), White (6.7:100,000; 95% CI 6.5–6.9), and Asian/Pacific Islander patients (6.6:100,000; 95% CI 6.4–6.9). AI/ANs had the second highest incidence of anal (2.1:100,000; 95% CI 1.5–2.8), penile (1.1:100,000; 95% CI 0.6, 2.1), and vulvar (2:100,000; 95% CI 1.3–3.1) cancer compared to other ethnicities. White patients had the highest incidence of anal cancer (2.9:100,000; 95% CI 2.8–3) in females, while Black patients had the highest in males (2.3:100,000; 95% CI 2–2.6), Hispanic patients had the highest incidence of penile cancer (1.3:100,000; 95% CI 1.2–1.4), and White patients had the highest incidence of vulvar cancer (2.4:100,000; 95% CI 2.3–2.5). The incidence of vaginal cancer was the same for AI/ANs (0.9:100,000; 95% CI 0.4–1.6) and Black patients (0.9:100,000; 95% CI 0.8–1.1), followed by White (0.7:100,000 95% CI 0.6–0.7) and Hispanic patients (0.7:100,000; 95% CI 0.6–0.8).

## 4. Discussion

With this study, we examined the burden of HNC in the AI/AN population in California. We then explored the incidence of HPV+ lip, oral cavity, and pharynx cancers between 2009 and 2018 and other HNCs between 2016 and 2018. HPV status was not collected for the soft palate, pharyngeal tonsil, and tongue base until 2016. Pharyngeal cancers (oropharynx, nasopharynx, hypopharynx, and other pharyngeal sites) were grouped in oral cavity and pharynx until 2016. We found that AI/AN and White patients had the highest burden of late stage HNC (AI/AN 6.3:100,000; 95% CI 5.3–7.4, White 5.8:100,000; 95% CI 5.7–5.9) and HPV+ lip, oral cavity, and pharynx HNC (AI/AN 0.9:100,000; 95% CI 0.6–1.4, White 1.1:100,000; 95% CI 1–1.1) compared to all ethnicities or races. Additionally, AI/ANs had a decreased 5-year survival rate compared to White patients (AI/ANs 59.9%; 95% CI 51.9–67.0% and White 67.7%; 95% CI 67.00–68.50%). The incidence of HNC in former and current tobacco users was highest in AI/AN patients compared to other ethnicities. Finally, the rates of overall HPV+ HNC from 2016 to 2018 were 1.3 to 4.5 times higher in the AI/AN population than Black, Hispanic, and Asian/Pacific Islander patients.

For the years 2016 to 2018, the incidence of HPV+ oropharyngeal cancer in AI/ANs (1.1:100,000; 95% CI 0.5–2.2) was higher than observed in Asian/Pacific Islanders, Hispanic, and Black ethnicities in California. The CDC similarly found a high proportion of HPV+ oropharyngeal HNC from 2013 to 2017 in the AI/AN population in the Pacific Coast region; 81% of the total HPV-associated cancers in AI/AN males (12.7:100,000) was oropharyngeal cancer [1,20,21]. This disparity underscores the importance of understanding why AI/AN patients in California have a higher incidence of HPV+ HNC. The difference in rates could be due to differences in sex practices such as oral sex, oro-anal contact, number of oral sexual partners, barrier contraceptive usage, age, and gender, all of which play a role in increased HPV transmission [22,23]. However, this information was not available in the CCR. The higher incidence should therefore serve as an immediate focus for healthcare vaccination policy, in congruence with increased education about HPV+ HNC, to impact future cancer diagnosis. FDA-approved HPV vaccines have been found to be successful at preventing HPV+ HNC when administered prior to HPV exposure [5,24]. As of 2019, in the United States, 56.8% of female and 51.8% of male adolescents, ages 13–17, completed the HPV vaccine series compared to 61.5% and 51.4% in California [25]. Interestingly, a study in Michigan found that from 2006 to 2015, HPV vaccination completion rates were higher in the AI/AN cohort compared to White patients [26]. From the Indian Health Service (IHS) clinics in California who submitted quarterly reports for 13- to 17-year-old males and females in 2021, there was a trend that HPV vaccination in California was lower than IHS national average with vaccine initiation 19–29% lower, second dose 26–29% lower, and third dose 7–26% lower [27,28,29]. AI/AN children are eligible for the Vaccines for Children Program (VFC) as defined by the Indian Health Care Improvement Act and can receive vaccines at no cost through VFC-enrolled doctors [30]. Future work should elucidate current yearly vaccination uptake patterns in vaccine-eligible AI/AN populations both at IHS and non-IHS facilities to help reduce oral HPV infection rates.

Our work also supports previous findings that revealed disparities in survival rates of AI/ANs diagnosed with HNC [10,11]. The survival rates may have been affected by higher tobacco use in AI/AN compared to other races and ethnicities. Tobacco usage can be attributed to the commercial tobacco industry’s targeting of AI/AN communities, leading to increased smoking prevalence as well as smoking-related diseases compared to other ethnicities or races [31,32]. In addition to the promotional and industry policy interventions to address the disparities, public health efforts should continue to work with tribes to denormalize recreational tobacco use.

It has been reported that HNC amongst AI/AN populations is often not diagnosed until later stages of the disease [4,10,11,33,34]. Our findings confirm and extend these observations, showing increased presentation of advanced stage HNC in AI/ANs in California. It is known that early detection of HNC leads to better prognosis [35]. Earlier detection and treatment of HNC can be improved by increasing provider surveillance across all races and ethnicities. Engaging AI/AN communities in all data-related processes and assessment of biases inherent in cancer surveillance is crucial to ensure that interventions are effective and culturally sensitive [36]. This will help elucidate multifactorial conditions contributing to disparities in HNC incidence and prognosis, such as socioeconomic status, exposure to tobacco and alcohol products, geographic distance from a cancer center, and health insurance status.

To our knowledge, this is the first study in California to reveal the disparity of HNC in the AI/AN population. Although this study used the most accurate and current data for cancer incidence in AI/AN populations in California, it has limitations. First, the AI/AN population, our population of interest, diagnosed with HNC in California was small compared to other races and ethnicities. This limited our ability to detect significant differences in the incidence of HNCs and interpretability of HPV-related data. Second, the California Cancer Registry only started collecting information on HPV status for all HNC sites since 2016. Therefore, we are limited in the number of cases where HPV status was known. Third, traditionally, the Veteran’s Health Administration (VA) hospitals have been a critical source of data for cancers diagnosed amongst veterans who are eligible to receive care from these facilities. Since 2007, the VA enacted new restrictions for the submission of cancer cases to central cancer registries. The missing VA cases may impact the incidence rates and trends as VA cancers account for 3 to 8 percent of national cancer statistics. Lastly, we were unable to include information on alcohol status, as it is not currently collected by the state registry. This is one of the most common risk factors of HNC.

## 5. Conclusions

In summary we found that AI/AN populations in California have a higher burden of late-stage HNC including HPV+ HNC compared to other populations. This underscores the racial disparities existing in the causes, presentation, and survival rate of HNC in AI/AN populations in California. Future studies should focus on identifying factors contributing to late-stage diagnosis and unequal burden of HNC outcomes. Efforts should be made to understand current HPV vaccination patterns for vaccine-eligible individuals and screening patterns in vaccine-ineligible individuals in the AI/AN population compared to other races and ethnicities in California. Additionally, future studies should elucidate how to best address increased tobacco use among California tribes. Creating culturally informed and community-based interventions will help reduce these disparities in HNC incidence in the population.

## Figures and Tables

**Table 1:** Age-adjusted incidence rates ^1^ of head and neck cancers ^2^, by race/ethnicity, sex, age at diagnosis, and tobacco use, 2009–2018 CA.

Demographics and Tobacco History	Non-Hispanic White		Non-Hispanic Black		Hispanic		Asian/Pacific Islander		American Indian/Alaska Native	
	Incidence Rates (CI)	N	Incidence Rates (CI)	N	Incidence Rates (CI)	N	Incidence Rates (CI)	N	Incidence Rates (CI)	N
Total	15.5 (15.3, 15.7)	34,448	12 (11.6, 12.5)	2946	8.3 (8.1, 8.5)	7959	9.1(8.8, 9.3)	5616	14.2 (12.6, 15.9)	320
Sex										
Male	23.6 (23.3, 23.9)	25,014	18.7 (17.8, 19.5)	2071	12.8 (12.5, 13.2)	5561	13.5 (13.1, 14)	3728	22 (19.1, 25.1)	233
Female	8.2 (8, 8.3)	9434	6.7 (6.2, 7.1)	875	4.6 (4.4, 4.8)	2398	5.6 (5.3, 5.8)	1888	7.4 (5.8, 9.2)	87
Age at Diagnosis										
<20	0.3 (0.2, 0.4)	90	0.4 (0.3, 0.6)	28	0.2 (0.2, 0.3)	110	0.3 (0.2, 0.4)	41	^	^
20–29	0.9 (0.7, 1)	172	0.9 (0.6, 1.2)	33	0.7 (0.6, 0.9)	187	1.3 (1.1, 1.6)	115	^	^
30–39	3 (2.8, 3.3)	550	2.2 (1.7, 2.8)	70	1.7 (1.5, 1.9)	367	4.3 (3.8, 4.7)	374	3.9 (1.9, 7.2)	10
40–49	10.7 (10.2, 11.1)	2281	9.3 (8.3, 10.4)	310	4.8 (4.5, 5.2)	942	9.1 (8.5, 9.8)	781	12.9 (9.1, 17.1)	38
50–59	31.4 (30.7, 32.1)	7872	25 (23.4, 26.8)	833	13.7 (13.1, 14.3)	1939	16.8 (15.9, 17.8)	1293	26.4 (21, 32.8)	82
60–69	51.7 (50.7, 52.7)	10,513	43.2 (40.4, 46.1)	913	25.7 (24.5, 26.8)	1986	24.7 (23.4, 26.1)	1368	45.1 (36.6, 54.9)	99
70–79	64.6 (63.2, 66.1)	7641	45.8 (41.9, 50)	509	39.6 (37.6, 41.6)	1504	32.8 (30.8, 34.9)	1005	49.8 (37.2, 65.3)	53
80+	64.4 (62.7, 66.2)	5329	41.9 (36.9, 47.4)	250	43.2 (40.4, 46)	924	34.7 (32.1, 37.5)	639	67.2 (46.5, 93.9)	34
Tobacco Use										
Never Used	2.5 (2.4, 2.5)	5351	1.4 (1.3, 1.6)	345	1.4 (1.4, 1.5)	1447	2.2 (2, 2.3)	1316	2.2 (1.6, 3)	46
Current Use	1.5 (1.5, 1.6)	3431	1.9 (1.7, 2.1)	496	0.6 (0.6, 0.7)	659	0.5 (0.4, 0.5)	316	1.8 (1.3, 2.5)	45
Former Use ^3^	2.9 (2.8, 3)	6557	1.9 (1.7, 2.1)	448	1.4 (1.3, 1.5)	1255	1.3 (1.2, 1.4)	816	3.2 (2.5, 4.1)	73
Unknown	5.9 (5.8, 6)	13,073	4.5 (4.2, 4.8)	1080	3.5 (3.4, 3.6)	3288	3.7 (3.5, 3.8)	2265	4.9 (4, 6)	109

^1^ Rates are per 100,000 and age-adjusted to the 2000 US Std Population (19 age groups—Census P25-1130) standard; Confidence intervals (Tiwari mod) are 95% for rates. ^2^ Head and neck cancers are comprised of diagnosed malignant cancers of the oral cavity, pharynx, nasopharynx, oropharynx, hypopharynx, larynx, paranasal sinuses and nasal cavity, and salivary glands. ^3^ Note: Former tobacco use is defined by having quit within one year of date of diagnosis, having quit more than one year prior to date of diagnosis, or unknown when quit.

**Table 2:** Age-adjusted incidence rates ^1^ of head and neck cancers ^2^, by race/ethnicity, stage at diagnosis, 5-year survival rate ^3^, and sex, 2009–2018 CA.

Staging	Non-Hispanic White		Non-Hispanic Black		Hispanic		Asian/Pacific Islander		American Indian/Alaska Native	
	Incidence Rates (CI)	N	Incidence Rates (CI)	N	Incidence Rates (CI)	N	Incidence Rates (CI)	N	Incidence Rates (CI)	N
Stage										
Male and Female										
Stage I	3.6 (3.5, 3.6)	7812	2 (1.8, 2.2)	469	1.7 (1.6, 1.8)	1621	1.8 (1.7, 1.9)	1102	2.4 (1.8, 3.2)	55
Stage II	1.7 (1.7, 1.8)	3784	1.4 (1.2, 1.5)	335	1 (0.9, 1)	901	1.4 (1.3, 1.5)	827	1.6 (1.1, 2.2)	37
Stage III	2.1 (2, 2.1)	4604	1.9 (1.7, 2.1)	454	1.2 (1.1, 1.3)	1110	1.5 (1.4, 1.6)	941	1.8 (1.2, 2.4)	40
Stage IV	5.8 (5.7, 5.9)	13,012	5.2 (4.9, 5.5)	1291	3.1 (3, 3.2)	3082	3.2 (3, 3.3)	1974	6.3 (5.3, 7.4)	144
All Stages	13.2 (13, 13.3)	29,212	10.4 (10, 10.8)	2549	7 (6.8, 7.2)	6714	7.8 (7.6, 8.1)	4844	12 (10.6, 13.6)	276
Male										
Stage I	5 (4.9, 5.2)	5246	3 (2.7, 3.4)	312	2.5 (2.4, 2.7)	1032	2.4 (2.2, 2.6)	649	3.4 (2.3, 4.8)	35
Stage II	2.5 (2.4, 2.6)	2598	1.9 (1.6, 2.1)	206	1.4 (1.3, 1.5)	581	1.9 (1.8, 2.1)	523	2.4 (1.6, 3.6)	27
Stage III	3.2 (3.1, 3.3)	3388	3 (2.7, 3.4)	328	1.9 (1.7, 2)	778	2.3 (2.1, 2.5)	639	2.5 (1.6, 3.7)	27
Stage IV	9.5 (9.3, 9.7)	10,214	8.4 (7.9, 9)	968	5.1 (4.9, 5.4)	2332	5.1 (4.8, 5.3)	1417	10.2 (8.3, 12.4)	111
All Stages	20.2 (19.9, 20.5)	21,446	16.3 (15.5, 17.1)	1814	10.9 (10.5, 11.1)	4723	11.7 (11.3, 12.1)	3228	18.5 (15.9, 21.4)	200
Female										
Stage I	2.3 (2.2, 2.4)	2566	1.2 (1, 1.4)	157	1.1 (1, 1.2)	589	1.4 (1.2, 1.5)	453	1.6 (1, 2.5)	20
Stage II	1 (1, 1.1)	1186	1 (0.8, 1.2)	129	0.6 (0.6, 0.7)	320	0.9 (0.8, 1)	304	0.9 (0.4, 1.7)	10
Stage III	1 (1, 1.1)	1216	0.9 (0.8, 1.1)	126	0.6 (0.6, 0.7)	332	0.9 (0.8, 1)	302	1.1 (0.5, 1.9)	13
Stage IV	2.4 (2.3, 2.5)	2798	2.4 (2.2, 2.7)	323	1.5 (1.3, 1.6)	750	1.6 (1.5, 1.8)	557	2.9 (1.9, 4.1)	33
All Stages	6.8 (6.6, 6.9)	7766	5.6 (5.2, 6)	735	3.8 (3.7, 4)	1991	4.8 (4.5, 5)	1616	6.4 (5, 8.1)	76
Mortality										
Male and Female	3.7 (3.6, 3.8)	8606	4 (3.8, 4.3)	957	2.3 (2.2, 2.4)	1994	2.6 (2.5, 2.8)	1601	2.9 (2.2, 3.8)	65
Male	5.8 (5.7, 5.9)	6180	6.6 (6.1, 7.2)	687	3.8 (3.6, 4.1)	1450	4.2 (4, 4.5)	1127	4.6 (3.3, 6.2)	48
Female	1.9 (1.8, 2)	2426	2.1 (1.8, 2.4)	270	1.2 (1.1, 1.3)	544	1.4 (1.3, 1.5)	474	1.5 (0.9, 2.5)	17
5 Year Survival Rate ^4^										
Male and Female	67.7% (67.00%, 68.50%)		55.95% (53.30%, 58.40%)		63.7% (62.2%, 65.30%)		66.4% (64.60%, 68.20%)		59.9% * (51.9% *, 67.0% *)	
Male	67.8% (66.90%, 68.60%)		56.4% (53.30%, 59.40%)		62.2% (60.30%, 64.10%)		63.8% (61.6%, 66.00%)		62.6% * (53.6%, 70.3% *)	
Female	67.7% (66.20%, 69.20%)		54.5% * (49.6% *, 59.0% *)		67.5% (64.60%, 70.30%)		71.9% (68.80%, 74.80%)		49.0% * (31.0% *, 64.7% *)	

^1^ Rates are per 100,000 and age-adjusted to the 2000 US Std Population (19 age groups—Census P25-1130) standard; Confidence intervals (Tiwari mod) are 95% for rates. ^2^ Head and neck cancers are comprised of diagnosed malignant cancers of the oral cavity, pharynx, nasopharynx, oropharynx, hypopharynx, larynx, paranasal sinuses and nasal cavity, and salivary glands. ^3^ Actuarial method. Ederer II method used for cumulative expected. ^4,^*: The relative cumulative survival increased from a prior interval and has been adjusted.

**Table 3:** Age-adjusted incidence rates ^1^ of lip, oral cavity, and pharynx cancer, by race/ethnicity and HPV status ^2^, 2009–2018 CA.

HPV Status	Non-Hispanic White		Non-Hispanic Black		Hispanic		Asian/Pacific Islander		American Indian/Alaska Native	
	Incidence Rates (CI)	N	Incidence Rates (CI)	N	Incidence Rates (CI)	N	Incidence Rates (CI)	N	Incidence Rates (CI)	N
Negative	4.9 (4.8, 5)	10,664	2.8 (2.5, 3)	662	2.3 (2.2, 2.4)	2215	2.8 (2.7, 2.9)	1719	3.5 (2.7, 4.4)	78
Positive	1.1 (1, 1.1)	2377	0.8 (0.7, 0.9)	202	0.4 (0.4, 0.5)	443	0.6 (0.5, 0.6)	354	0.9 (0.6, 1.4)	23
Unknown	2.1 (2, 2.1)	4625	1.8 (1.7, 2)	459	1.2 (1.1, 1.2)	1136	1.5 (1.4, 1.6)	916	2 (1.4, 2.7)	41

^1^ Rates are per 100,000 and age-adjusted to the 2000 US Std Population (19 age groups—Census P25-1130) standard; Confidence intervals (Tiwari mod) are 95% for rates. ^2^ HPV status was collected as defined by the CS Collaborative Stage Data Collecting System, version 02.05 schemas.

**Table 4:** Age-adjusted incidence rates ^1^ of hypopharynx, nasopharynx, oropharynx, soft palate, pharyngeal tonsil, Tongue base, and other pharyngeal cancer, by race/ethnicity and HPV status ^2^, 2016–2018 CA.

HPV Status	Non-Hispanic White		Non-Hispanic Black		Hispanic		Asian/Pacific Islander		American Indian/Alaska Native	
	Incidence Rate (CI)	N	Incidence Rate (CI)	N	Incidence Rate (N)	N	Incidence Rate (CI)	N	Incidence Rate (CI)	N
Overall ^3^										
Negative	1.4 (1.3, 1.4)	934	0.8 (0.6, 1)	67	0.7 (0.6, 0.8)	220	1.1 (1, 1.3)	241	1.1 (0.5, 2.3)	9
Positive	2.8 (2.7, 3)	1952	1.4 (1.2, 1.7)	117	0.7 (0.6, 0.8)	263	0.6 (0.5, 0.7)	132	2.7 (1.6, 4.2)	21
Unknown	3.2 (3.1, 3.4)	2225	2.5 (2.1, 2.8)	192	2 (1.8, 2.1)	645	2.8 (2.6, 3.1)	586	1.9 (1, 3.2)	15
Hypopharynx										
Negative	0.1 (0.1, 0.1)	82	0.1 (0, 0.2)	8	0 (0, 0.1)	15	0.1 (0,1, 0.1)	19	^	^
Positive	0.1 (0, 0.1)	40	^	^	0 (0, 0)	6	0 (0, 0.1)	6	^	^
Unknown	0.2 (0.1, 0.2)	111	0.2 (0.1, 0.3)	17	0.1(0.1, 0.1)	34	0.1(0.1, 0.2)	25	^	^
Nasopharynx										
Negative	0.1 (0, 0.1)	36	0.1 (0, 0.2)	6	0 (0, 0.1)	14	0.4 (0.3, 0.5)	83	^	^
Positive	0 (0, 0.1)	33	0.1 (0, 0.2)	8	0 (0, 0)	5	0.2 (0.1, 0.2)	33	^	^
Unknown	0.2 (0.1, 0.2)	102	0.3 (0.2, 0.4)	20	0.1 (0.1, 0.2)	50	1 (0.9, 1)	205	^	^
Oropharynx										
Negative	0.2 (0.2, 0.3)	155	0.1(0, 0.2)	7	0.1(0.1, 0.2)	46	0.1(0, 0.1)	16	^	^
Positive	1.4 (1.3, 1.5)	939	0.8 (0.6, 1)	65	0.5 (0.4, 0.5)	165	0.2 (0.2, 0.3)	49	1.8 (1, 3.1)	14
Unknown	0.6 (0.6, 0.7)	428	0.6 (0.4, 0.8)	48	0.4 (0.4, 0.6)	175	0.3 (0.2, 0.4)	63	^	^
Soft Palate										
Negative	0.1 (0, 0.1)	35	^	^	^	^	^	^	^	^
Positive	0 (0, 0)	19	^	^	^	^	^	^	^	^
Unknown	0.1 (0, 0.1)	36	^	^	^	^	^	^	^	^
Pharyngeal Tonsil										
Negative	^	^	^	^	^	^	0.1 (0, 0.1)	13	^	^
Positive	^	^	^	^	^	^	^	^	^	^
Unknown	0 (0, 0)	10	^	^	^	^	0.1 (0, 0.1)	15	^	^
Tongue Base										
Negative	0.2 (0.1, 0.2)	119	0.2 (0.1, 0.3)	17	0.1(0.1, 0.1)	29	0.1(0, 0.1)	15	^	^
Positive	1.1 (1, 1.2)	775	0.4 (0.2, 0.5)	30	0.2 (0.1, 0.2)	64	0.1 (0.1, 0.2)	21	0.9 (0.3, 1.9)	7
Unknown	0.5 (0.5, 0.6)	371	0.2 (0.1, 0.3)	14	0.2 (0.2, 0.3)	77	0.1 (0.1, 0.2)	21	^	^
Other Pharyngeal										
Negative	0 (0, 0)	23	^	^	0 (0, 0)	5	^	^	^	^
Positive	0 (0,0.1)	25	^	^	^	^	^	^	^	^
Unknown	0 (0, 0)	45	0.1 (0, 0.1)	5	0 (0, 0.1)	13	0.1 (0, 0)	10	^	^

^1^ Rates are per 100,000 and age-adjusted to the 2000 US Std Population (19 age groups—Census P25-1130) standard; Confidence intervals (Tiwari mod) are 95% for rates. ^2^ HPV status was collected as defined by the CS Collaborative Stage Data Collecting System, version 02.05 schemas. ^3^ Overall incidence rate also includes lip, oral cavity, and pharynx HNC for the years 2016–2018.

**Table 5:** Age-adjusted incidence rates ^1^ of cervical, anal, penile, vaginal, and vulvar ^2^, 2009–2018 CA.

Cancer Type	Non-Hispanic White		Non-Hispanic Black		Hispanic		Asian/Pacific Islander		American Indian/Alaska Native	
	Incidence Rate (CI)	N	Incidence Rate (CI)	N	Incidence Rate (CI)	N	Incidence Rate (CI)	N	Incidence Rate (CI)	N
Cervical	6.7 (6.5, 6.9)	5670	7.7 (7.2, 8.2)	946	9.1 (8.9, 9.4)	5545	6.6 (6.4, 6.9)	2216	13 (10.8, 15.4)	134
Anal										
Male and Female	2.4 (2.4, 2.5)	5450	2 (1.8, 2.2)	496	1.1(1, 1.2)	1085	0.5 (0.4, 0.6)	310	2.1 (1.5, 2.8)	50
Male	1.9 (1,8, 2)	2001	2.3 (2, 2.6)	265	0.9 (0.8, 1)	417	0.4 (0.4, 0.5)	126	2.1 (1.3, 3.2)	24
Female	2.9 (2.8, 3)	3449	1.8 (1.5, 2)	231	1.3 (1.2, 1.4)	668	0.5 (0.5, 0.6)	184	2 (1.3, 3)	26
Penile	0.7 (0.6, 0.7)	669	0.6 (0.5, 0.8)	61	1.3 (1.2, 1.4)	557	0.5 (0.4, 0.5)	116	1.1 (0.6, 2.1)	11
Vaginal	0.7 (0.6, 0.7)	820	0.9 (0.8, 1.1)	119	0.7 (0.6, 0.8)	345	0.5 (0.4, 0.5)	164	0.9 (0.4, 1.6)	10
Vulvar	2.4 (2.3, 2.5)	2909	1.6 (1.4, 1.8)	205	1.7 (1.5, 1.8)	789	0.8 (0.7, 0.9)	279	2 (1.3, 3.1)	25

^1^ Rates are per 100,000 and age-adjusted to the 2000 US Std Population (19 age groups—Census P25-1130) standard; Confidence intervals (Tiwari mod) are 95% for rates. ^2^ HPV-related cancers.

## Data Availability

The data presented in this study are available on request from the corresponding author.

## References

[1] Disparities. https://www.ihs.gov/newsroom/factsheets/disparities/.

[2] Melkonian S.C., Jim M.A., Haverkamp D., Wiggins C.L., McCollum J., White M.C., Kaur J.S., Espey D.K. (2019). Disparities in cancer incidence and trends among American Indians and Alaska natives in the United States, 2010–2015. Cancer Epidemiol. Biomark. Prev..

[3] Zavala V.A., Bracci P.M., Carethers J.M., Carvajal-Carmona L., Coggins N.B., Cruz-Correa M.R., Davis M., de Smith A.J., Dutil J., Figueiredo J.C. (2021). Cancer health disparities in racial/ethnic minorities in the United States. Br. J. Cancer.

[4] Siegel R.L., Miller K.D., Fuchs H.E., Jemal A. (2021). Cancer Statistics, 2021. CA Cancer J. Clin..

[5] Johnson D.E., Burtness B. (2020). Head and neck squamous cell carcinoma. Nat. Rev. Dis. Prim..

[6] Shavers V.L., Harlan L.C., Winn D., Davis W.W. (2003). Racial/ethnic patterns of care for cancers of the oral cavity, pharynx, larynx, sinuses, and salivary glands. Cancer Metastasis Rev..

[7] Thompson-Harvey A., Yetukuri M., Hansen A.R., Simpson M.C., Adjei Boakye E., Varvares M.A., Osazuwa-Peters N. (2020). Rising incidence of late-stage head and neck cancer in the United States. Cancer.

[8] Centers for Disease Control United States Cancer Statistics: Data Visualizations. https://gis.cdc.gov/Cancer/USCS/#/AtAGlance/.

[9] Mahal B.A., Inverso G., Aizer A.A., Bruce Donoff R., Chuang S.K. (2014). Impact of African-American race on presentation, treatment, and survival of head and neck cancer. Oral Oncol..

[10] Dwojak S.M., Sequist T.D., Emerick K., Deschler D.G. (2013). Survival differences among American Indians/Alaska natives with head and neck squamous cell carcinoma. Head Neck.

[11] Morris C., Movsisyan A., Hofer B., Parikh-Patel A., Kizer K. (2019). Cancer Burden among Native Americans in California.

[12] World Health Organization (2013). International Classification of Diseases for Oncology (ICD-O).

[13] Norris T., Vines P.L., Hoeffel E.M. (2012). The American Indian and Alaska Native Population: 2010. 2010 Census Briefs.

[14] Dwojak S., Bhattacharyya N. (2017). Racial disparities in preventable risk factors for head and neck cancer. Laryngoscope.

[15] United States Census Bureau QuickFacts: California. https://www.census.gov/quickfacts/fact/table/CA/RHI325219#RHI325219.

[16] California Cancer Registry. https://www.ccrcal.org/learn-about-ccr/.

[17] National Cancer Institute’s Surveillance, E. and E.R.P Head and Neck with HPV Status Database (2010–2016). https://seer.cancer.gov/seerstat/databases/hpv/index.html.

[18] National Cancer Institute’s Surveillance, E. and E.R.P EOD Data: Human Papilloma Virus (HPV) Status. https://staging.seer.cancer.gov/eod_public/input/1.1/gum/seer_ssf1/?breadcrumbs=(~schema_list~),(~view_schema~,~gum~.

[19] California Cancer Registry California Department of Public Health SEER*Stat Database: Incidence–California, Dec 2020 (1998–2018), 03/03/2021; Benchmarked 1988–1989 DOF Population Estimates, 6/12/2006; NCHS Population Estiamtes 1990–2018. www.ccrcal.org.

[20] Melkonian S.C., Henley S.J., Senkomago V., Thomas C.C., Jim M.A., Apostolou A., Saraiya M. (2020). Cancers Associated with Human Papillomavirus in American Indian and Alaska Native Populations—United States, 2013–2017. MMWR. Morb. Mortal. Wkly. Rep..

[21] Melkonian S.C., Weir H.K., Jim M.A., Preikschat B., Haverkamp D., White M.C. (2021). Incidence of and Trends in the Leading Cancers with Elevated Incidence among American Indian and Alaska Native Populations, 2012–2016. Am. J. Epidemiol..

[22] Shah A., Malik A., Garg A., Mair M., Nair S., Chaturvedi P. (2017). Oral sex and human papilloma virus-related head and neck squamous cell cancer: A review of the literature. Postgrad. Med. J..

[23] Nguyen N.P., Nguyen L.M., Thomas S., Hong-Ly B., Chi A., Vos P., Karlsson U., Vinh-Hung V. (2016). Oral sex and oropharyngeal cancer: The role of the primary care physicians. Medicine.

[24] Gillison M.L., Chaturvedi A.K., Lowy D.R. (2008). HPV prophylactic vaccines and the potential prevention of noncervical cancers in both men and women. Cancer.

[25] Walker T.Y., Elam-Evans L.D., Yankey D., Markowitz L.E., Williams C.L., Fredua B., Singleton J.A., Stokley S. (2019). National, Regional, State, and Selected Local Area Vaccination Coverage Among Adolescents Aged 13–17 Years—United States, 2018. MMWR. Morb. Mortal. Wkly. Rep..

[26] Kashani B.M., Tibbits M., Potter R.C., Gofin R., Westman L., Watanabe-Galloway S. (2019). Human Papillomavirus Vaccination Trends, Barriers, and Promotion Methods Among American Indian/Alaska Native and Non-Hispanic White Adolescents in Michigan 2006–2015. J. Community Health.

[27] Gopalani S.V., Sedani A.E., Janitz A.E., Clifton S.C., Stoner J., Peck J., Comiford A., Salvatore A.L., Campbell J. (2020). HPV vaccination and Native Americans: Protocol for a systematic review of factors associated with HPV vaccine uptake among American Indians and Alaska Natives in the USA. BMJ Open.

[28] Centers for Disease Control and Prevention TeenVax View. https://www.cdc.gov/vaccines/imz-managers/coverage/teenvaxview/index.html.

[29] Indian Health Service National Immunization Reporting System Reports. https://www.ihns.gov/nonmedicalprograms/ihpes/Immunizations/index.cfm?module=immunizations&option=home.

[30] Centers for Disease Control and Prevention Vaccines for Children Program. https://www.cdc.gov/vaccines/programs/vfc/parents/qa-detailed.html.

[31] Creamer M.R., Wang T.W., Babb S., Cullen K.A., Day H., Willis G., Jamal A., Neff L. (2019). Tobacco Product Use and Cessation Indicators Among Adults—United States, 2018. MMWR. Morb. Mortal. Wkly. Rep..

[32] Lempert L.K., Glantz S.A. (2019). Tobacco industry promotional strategies targeting American Indians/Alaska natives and exploiting tribal sovereignty. Nicotine Tob. Res..

[33] Espey D.K., Wu X.C., Swan J., Wiggins C., Jim M.A., Ward E., Wingo P.A., Howe H.L., Ries L.A.G., Miller B.A. (2007). Annual report to the nation on the status of cancer, 1975–2004, featuring cancer in American Indians and Alaska Natives. Cancer.

[34] Reichman M.E., Kelly J.J., Kosary C.L., Coughlin S.S., Jim M.A., Lanier A.P. (2008). Incidence of cancers of the oral cavity and pharynx among American Indians and Alaska Natives, 1999–2004. Cancer.

[35] Vokes E.E., Weichselbaum R.R., Lippman S.M., Hong W.K. (1993). Head and Neck Cancer. N. Engl. J. Med..

[36] Tatsuzaki H., Levin C.V. (2001). Quantitative status of resources for radiation therapy in Asia and Pacific region. Radiother. Oncol..

